# Comparative Analysis of Therapeutic Strategies in Post-Cardiotomy Cardiogenic Shock: Insight into a High-Volume Cardiac Surgery Center

**DOI:** 10.3390/jcm13072118

**Published:** 2024-04-05

**Authors:** B. Ufuk Baldan, Romy R. M. J. J. Hegeman, Nelleke M. J. P. Bos, Hans G. Smeenk, Robert J. M. Klautz, Patrick Klein

**Affiliations:** 1 Department of Cardiothoracic Surgery, Amsterdam University Medical Center, 1105 AZ Amsterdam, The Netherlands; 2 Department of Cardiothoracic Surgery, St. Antonius Hospital, 3435 CM Nieuwegein, The Netherlands; h.smeenk@antoniusziekenhuis.nl; 3 Bos Healthcare Consultancy, 1036 KM Amsterdam, The Netherlands

**Keywords:** post-cardiotomy cardiogenic shock (PCCS), extracorporeal membrane oxygenation (ECMO), Stage Classification Expert Consensus (SCAI)

## Abstract

**Background**: Post-cardiotomy cardiogenic shock (PCCS), which is defined as severe low cardiac output syndrome after cardiac surgery, has a mortality rate of up to 90%. No study has yet been performed to compare patients with PCCS treated by conservative means to patients receiving additional mechanical circulatory support with veno-arterial extracorporeal membrane oxygenation (ECMO). **Methods**: A single-center retrospective analysis from January 2018 to June 2022 was performed. **Results**: Out of 7028 patients who underwent cardiac surgery during this time period, 220 patients (3%) developed PCCS. The patients were stratified according to their severity of shock based on the Stage Classification Expert Consensus (SCAI) group. Known risk factors for shock-related mortality, including the vasoactive–inotropic score (VIS) and plasma lactate levels, were assessed at structured intervals. In patients treated additionally with ECMO (*n* = 73), the in-hospital mortality rate was 60%, compared to an in-hospital mortality rate of 85% in patients treated by conservative means (non-ECMO; *n* = 52). In 18/73 (25%) ECMO patients, the plasma lactate level normalized within 48 h, compared to 2/52 (4%) in non-ECMO patients. The morbidity of non-ECMO patients compared to ECMO patients included a need for dialysis (42% vs. 60%), myocardial infarction (19% vs. 27%), and cerebrovascular accident (17% vs. 12%). **Conclusions**: In conclusion, the additional use of ECMO in PCCS holds promise for enhancing outcomes in these critically ill patients, more rapid improvement of end-organ perfusion, and the normalization of plasma lactate levels.

## 1. Introduction

Post-cardiotomy shock (PCS) is a clinical condition that remains poorly defined in the literature but implies a state of circulatory failure after cardiac surgery, necessitating high dosages of vasoactive medication, inotropes, and occasionally, mechanical circulatory support (MCS) [1]. The incidence of PCS in adult patients undergoing elective cardiac surgery is 2% to 6%, and more severe stages of PCS are associated with in-hospital mortality rates up to 90% [2]. Post-cardiotomy cardiogenic shock (PCCS) is a form of PCS that is defined by severe low cardiac output syndrome (LCOS), referring to a clinical condition after cardiac surgery when the cardiac output is insufficient to meet the metabolic demands of the end organs [3].

The pathophysiology of PCCS involves a complex interplay of various factors. It often begins with damage to the myocardium during cardiac surgery, the use of cardiopulmonary bypass, or cardioplegic myocardial arrest [4]. Insufficient myocardial protection or residual valvular dysfunction severely aggravates the situation and increases myocardial stunning, myocardial ischemia, ischemia–reperfusion injury, and left and/or ventricular pressure and/or volume overload, causing LCOS [1,2,3]. Additionally, a cascade of events can be set off, including inflammatory responses, neurohormonal activation, and endothelial dysfunction. LCOS, especially in combination with hypotension because of the loss of peripheral vascular resistance in patients with preexistent compromised autoregulation, leads to reduced end-organ perfusion, end-organ failure, and ultimately, death [3]. The management of PCCS consists firstly of treatment of the underlying cardiac dysfunction, alongside hemodynamic stabilization, and support of end-organ hypoperfusion and dysfunction by volume resuscitation and pharmacological therapy (inotropes and/or vasopressors) [1,2,4]. MCS provides additional hemodynamic support by providing additional (forward) flow with or without the unloading of the right and/or left ventricle, depending on the type and canulation of different types of MCS [4].

The use and timing of different forms of MCS in PCCS, of which veno-arterial extracorporeal membrane oxygenation (ECMO) is currently the most widely used support system, is the subject of an ongoing debate, and many factors and, obviously, subjective choices play a role in its institution in these critically ill patients. ECMO restores myocardial and systemic perfusion and unloads the right ventricle instantaneously, thus potentially preventing further damage to the end organs. However, despite the large increase in its use over the past two decades, no improvement in the survival of patients in whom it has been used has been observed [4]. A collaborative initiative involving the European Association of Cardio-Thoracic Surgery (EACTS), the Society of Thoracic Surgeons (STS), the American Association for Thoracic Surgery (AATS), and Extracorporeal Life Support Organization (ELSO) published an expert consensus guideline for ECMO in PCCS [5]. Nevertheless, no clear indications and recommendations regarding the timing, ECMO settings, and patient characteristics were stated, besides the option to initiate ECMO in patients requiring multiple or high-dose inotropes during either the intraoperative weaning phase from the extracorporeal circulation or during the postoperative period. Given the heterogeneity of the PCCS population, defining clear indications for the use of ECMO is difficult.

While there have been many studies on the technical aspects and outcomes of ECMO in PCCS, no study has evaluated the treatment and outcomes of PCCS in general and conducted a comparative analysis between patients managed conservatively and patients who received additional ECMO [6,7,8,9,10]. An important step for a sensible comparison is the categorization of patients according to the severity of cardiogenic shock, incorporating a combination of physical findings, biomarkers, and hemodynamics as proposed by the Stage Classification Expert Consensus (SCAI) group [11,12]. Early identification of cardiac dysfunction and circulatory failure enables the timely administration of appropriate targeted and general therapy, potentially preventing further progression of PCCS, which leads to higher SCAI shock stages.

With this comparative analysis at a high-volume cardiac surgery center, we aim to provide additional insight into the treatment of PCCS at different severity stages of shock, with and without ECMO, and to identify prognostic predictors for survival.

## 2. Materials and Methods

A retrospective, single-center study was conducted at the St. Antonius Hospital (Nieuwegein, The Netherlands) between January 2018 and June 2022 to assess the occurrence and progression of cardiogenic shock in PCS patients, with and without ECMO. 

### 2.1. Primary Outcome and Secondary Outcomes

The primary objective was to assess survival across different stages of shock based on the SCAI classification and specifically between the ECMO and a non-ECMO group. Additionally, we examined differences within the ECMO subgroups, which consisted of intraoperative and postoperative ECMO, as well as early and late postoperative ECMO. The secondary objectives included postoperative left, right, or biventricular failure, cerebral vascular accidents (CVAs), acute myocardial infarction (AMI), rethoracotomies, kidney failure, dialysis, liver dysfunction, lactate and VIS normalization, and length of hospital and intensive care unit (ICU) stay.

### 2.2. Patient Selection

All patients who underwent primary cardiac surgery between January 2018 and June 2022 and met our inclusion criteria were manually reviewed to validate cardiogenic shock. No discrimination was made between elective and emergency procedures. The inclusion criteria were as follows: (1) cardiac surgery as a primary intervention, (2) simultaneous administration of noradrenaline and milrinone, (3) cumulative stay of more than 72 h in the ICU, and in the event of patient mortality, inclusion is based on the occurrence of death within 72 h postoperatively due to cardiac disease. Patients who initially underwent percutaneous coronary intervention (PCI) or primarily received ECMO therapy were excluded from this study. 

### 2.3. Definitions

PCCS was defined as the need for pharmacological therapy or devices to maintain a systolic blood pressure (SBP) above 90 mm Hg or a mean arterial pressure (MAP) above 60 mm Hg, along with a plasma lactate level exceeding 2 mmol/L, and with any postoperative echocardiographic evidence indicating deteriorated cardiac function [4,5,7,8,10,12]. Moreover, we have followed the definitions for secondary outcomes as outlined by the Dutch Heart Registry and shown in the Supplementary Materials, Table S1.

### 2.4. Stage Classification Expert Consensus (SCAI) Classification

**Figure 1:**
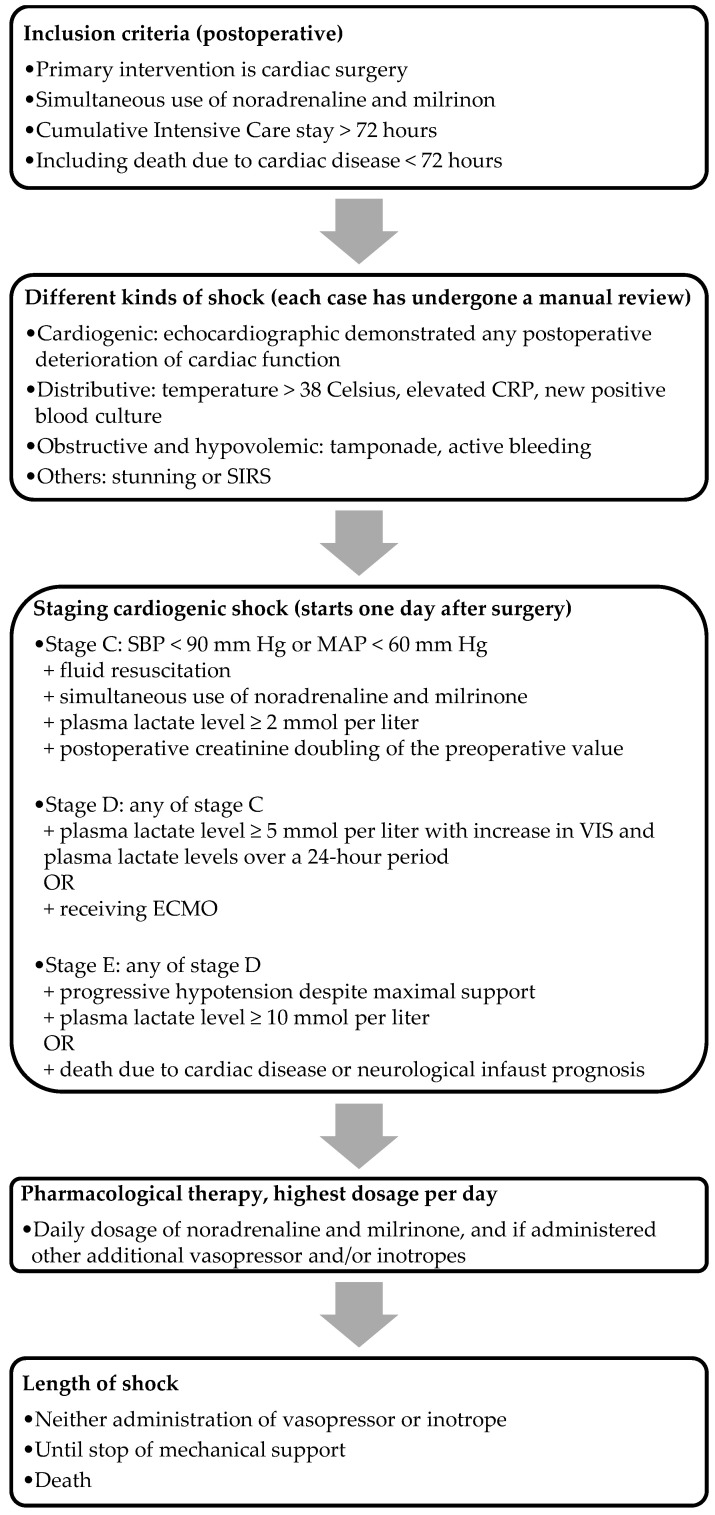
Flowchart of the inclusion method. Note: CRP, C-reactive protein; SIRS, systemic inflammatory response syndrome; ECMO, extracorporeal membrane oxygenation.

SCAI classification is a straightforward, clinically applicable classification tool incorporating physical findings, biomarkers, and hemodynamics [11]. We included the majority of the updated SCAI classification’s (2022) cutoff values, aligning with the modifications proposed by Kapur et al. [12]. The criteria for the stages, as illustrated in Figure 1*,* were used to assess the severity of PCCS. We focused exclusively on PCCS patients in stage C and onwards, excluding those in stages A and B. This decision was driven by the disrupted hemodynamics commonly observed postoperatively, and the fact that, by definition, these patients are not in cardiogenic shock. Stage C (i.e., classic cardiogenic shock) was characterized by postoperative creatinine doubling, the need for fluid administration, and at least one vasopressor and one inotrope to maintain an SBP above 90 mm Hg or an MAP above 60 mm Hg, along with an elevated plasma lactate level between 2 and 5 mmol/L. Patients who did not meet the criteria for stage C were classified under other etiologies. Patients met the criteria for stage D with the presence of each criterion of stage C, with the addition of a plasma lactate level between 5 and 10 mmol/L, along with an increase in both the VIS and plasma lactate levels over a 24 h period, or those who received ECMO. Stage E was defined using the criteria identical to those in stage D, with the additional requirement of a plasma lactate level of at least 10 mmol/L, or in patients in whom death was the result of cardiac pathology, including those with a neurological infaust prognosis. The staging for the ECMO group was determined just before the initiation of ECMO, while for the non-ECMO group, it corresponded to the nadir of their shock. For the analysis of non-ECMO versus ECMO, we only included PCCS patients in stages D and E in the non-ECMO group.

### 2.5. Data Collection

The patients’ demographic data, pre- and postoperative laboratory levels, surgical details, outcomes, and complication data were collected and obtained from their electronic medical records, which were kept in a centralized medical database. For the ECMO patients, we also evaluated the particular settings, duration, and variables at different time intervals. Additional data, including pharmacological therapy and postoperative echocardiographic findings, were collected using a manual search. 

### 2.6. Cardiac Deterioration

To determine postoperative primary left, right, or biventricular cardiac failure, we took the transthoracic echocardiographic (TTE) results obtained by the cardiologist and the preoperative echocardiographic records as well as the documented visual findings of the cardiothoracic surgeon from the rethoracotomy into account. If postoperative patients were not faring well, they underwent a TTE in the ICU. For instance, if it was detected that the left ventricle had deteriorated compared to the preoperative TTE, this decline was documented as primary left ventricular failure. Furthermore, the diagnosis of myocardial infarction was based on postoperative coronary angiogram findings, electrocardiogram (ECG) changes, elevated cardiac enzymes, and/or physical symptoms. 

### 2.7. Vasoactive–Inotropic Score and Plasma Lactate Level Normalization

The degree of hemodynamic instability leading to circulatory failure was assessed using the markers of the VIS and the plasma lactate levels [8,10,13,14,15,16]. Any adjustment or change in pharmacological therapy was automatically documented in the patient’s electronic medical record. The VIS represents a cumulative sum of all administered vasopressors and inotropes, and it was calculated as 100 × noradrenaline (μg/kg/min) + 10 × milrinone dose (μg/kg/min) + dopamine dose (μg/kg/min) + dobutamine dose (μg/kg/min) + 100 × adrenaline dose (μg/kg/min) + 10 × terlipressin dose (μg/min) + 10.000 × vasopressin dose (U/kg/min). However, the formula did not account for argipressin. Given that only argipressin, considered an equivalent to terlipressin, was utilized in our hospital, we did not modify the formula [13]. Throughout the duration of PCCS, we retrospectively extracted the highest daily administration of vasopressors and inotropes. Simultaneous administration was deemed valid if it was constantly and simultaneously given 24 h postoperatively. In case surgical intervention was necessary due to complications within the same admission, we reevaluated the patients using the same criteria after the intervention. 

During the period of PCCS, arterial blood gases were routinely taken every six hours, from which the plasma lactate value was determined. The normalization of plasma lactate was defined as the moment at which the plasma lactate level dropped below 2 mmol/L. 

### 2.8. Statistical Analysis

Continuous variables were compared using the independent t-test and Kruskal–Wallis test, while the Chi-square test was used for categorical variables. Kaplan–Meier analysis with additional log-rank tests was conducted to identify the variables associated with mortality in our PCCS group, particularly within the ECMO subgroups. Furthermore, we used logistic regression analyses to examine the association between preoperative, intraoperative, and postoperative variables and ECMO initiation. A *p*-value of less than 0.05 was considered statistically significant. All statistical analyses were performed using SPSS Statistics 26.0 (IBM Corp, Armonk, NY, USA). 

## 3. Results

### 3.1. Baseline Characteristics

Between January 2018 and June 2022, a total of 7.028 cardiac surgical procedures were performed. Postoperatively, 689 (10% ) patients met the inclusion criteria for shock (*p* < 0.001). A total of 220 patients (32%) were in PCCS (*p* < 0.001). The mean age of the overall cohort was 69 ± 9 years (*p* < 0.002), with 61% of the patients being male (*p* < 0.736) and a mean body mass index (BMI) of 26 ± 7 (*p* = 0.001). Significant differences were observed among the patients in different stages of shock, as seen in Table 1. Notable differences among the stages included a higher prevalence of diabetes mellitus in stage E (47%) compared to stage D (25%) and stage C (24%) (*p* < 0.005). Recent myocardial infarction was more prevalent in stage E (38%) than in stage D (36%) and stage C (21%) (*p* < 0.040). In general, as shown in Table 2, within the PCCS population, a relatively large proportion of patients had undergone more complex surgical procedures. Non-ECMO patients were older (mean age difference of 8 years) compared to ECMO patients (*p* < 0.001) and had a lower rate of previous cardiac surgery (13% vs. 29%, *p* = 0.043). Both groups showed comparable EuroSCORE II (10%, *p* < 0.766). Table 3 presents the baseline characteristics of non-ECMO and ECMO patients, and the Supplementary Materials, Table S2 shows the surgical data of these groups.

### 3.2. Reasons for Not Receiving ECMO

Reasons for not receiving ECMO (*n* = 44) among deceased patients in the non-ECMO group included being considered unsuitable for coronary revascularization or having no other therapeutic (surgical and non-surgical) options (44%, *p* < 0.001), poor preoperative clinical condition (22%, *p* < 0.001), sepsis development (20%, *p* = 0.002), or poor neurological outcomes (9%, *p* = 0.044). No reason for not receiving ECMO could be found in 2% (*p* = 0.323). 

### 3.3. Clinical Outcomes

The clinical outcomes of the different SCAI shock stages are presented in Table 4 and Table 5. In-hospital mortality increased from 44% at SCAI shock stage D to 94% at shock stage E (*p* < 0.001). Right-sided ventricular failure was 33% at shock stage C, 46% at shock stage D, and 24% at shock stage E (*p* < 0.038). At shock stage C, the patients showed an average hospital stay of 518 h; at shock stage D, it was 474 h, and at shock stage E 122 h (*p* < 0.001). It is important to mention that the non-ECMO and ECMO groups consisted only of patients at SCAI shock stages D and E, and none of the patients at SCAI shock stage C were included. Non-ECMO patients showed a mortality rate of 85% (*p* = 0.003), a postoperative need of dialysis rate of 42% (*p* = 0.047), and an ICU stay of 147 h (*p* = 0.019). ECMO patients showed a mortality rate of 60% (*p* = 0.003). In Table 6 more detailed clinical outcomes regarding the non-ECMO and ECMO groups are shown. Among the ECMO patients at shock stage D during ECMO initiation, 55% (*p* < 0.001) died. In the case of ECMO patients who progressed from shock stage D to shock stage E during ECMO, the mortality rate rose to 90% (*p* < 0.001). For PCCS patients already at shock stage E at the time of ECMO initiation, the mortality rate remained at 90% (*p* < 0.001). Furthermore, the ECMO group showed a postoperative need for a dialysis rate of 60% (*p* = 0.047) and an ICU stay of 308 h (*p* = 0.019). For more data regarding the ECMO settings, see Table 7.

### 3.4. Vasoactive–Inotropic Score and Plasma Lactate Level Normalization

**Figure 2:**
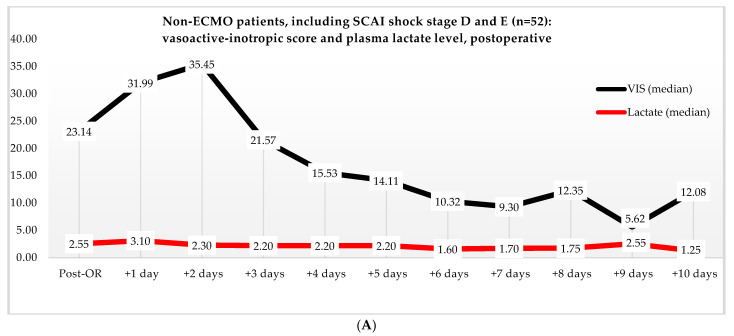

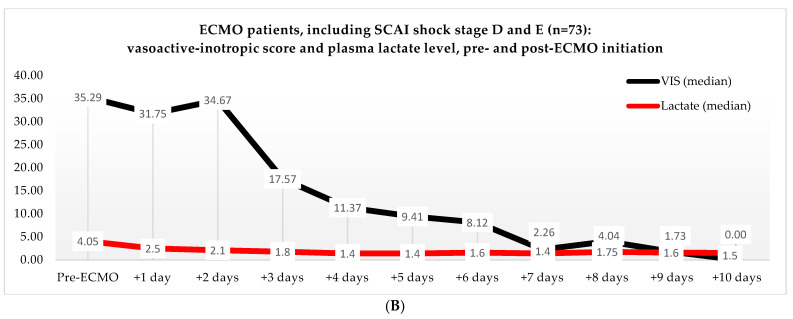
(**A**) Median VIS and plasma lactate levels after primary surgery of non-ECMO patients, including only shock stages D and E. Note: OR, operation room. (**B**) Median VIS and plasma lactate levels of the ECMO patients pre- and post-ECMO initiation.

**Figure 3:**
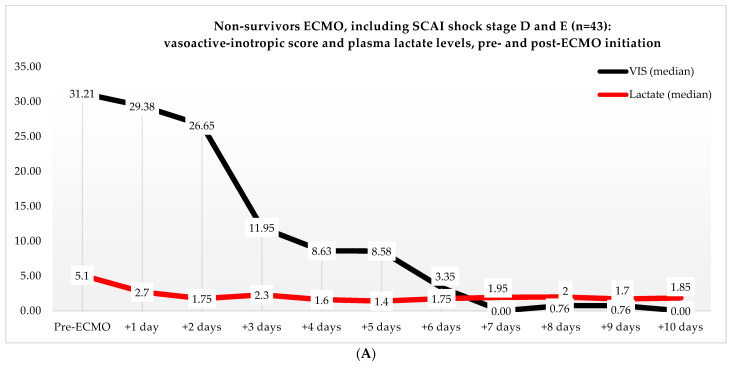

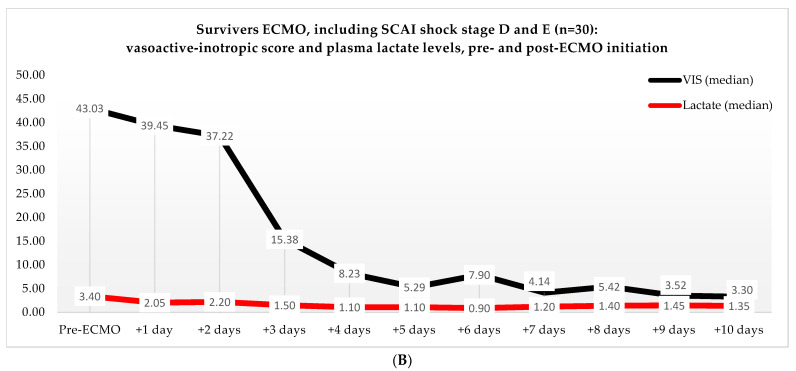
(**A**) Median VIS and plasma lactate levels of non-survivors in the ECMO group, pre- and post-ECMO initiation. (**B**) Median VIS and plasma lactate levels of survivors in the ECMO group, pre- and post-ECMO initiation.

Non-ECMO patients showed a faster normalization of the VIS (96 vs. 216 h) than the ECMO patients (*p* = 0.179). Postoperatively, the VIS of the non-ECMO patients peaked at 35.45 after two days. Additionally, in the ECMO group, after 12 h of ECMO initiation, a peak median VIS of 33.83 was observed, followed by a gradual decline. The non-ECMO patients had a longer median time to plasma lactate level normalization (70 vs. 41 h, *p* = 0.222), and fewer patients achieved plasma lactate level normalization within 48 h (6 vs. 28, *p* < 0.001) compared to the ECMO patients, as illustrated in Table 6 and Figure 2. In the non-ECMO group, non-survivors had a higher postoperative VIS than survivors (23.81 vs. 7.03, *p* = 0.043), and additionally, non-survivors had a maximum median VIS of 37.27 compared to 23.72 for survivors (*p* = 0.157), as shown in the Supplementary Materials, Figure S1. Furthermore, non-survivors exhibited a higher postoperative plasma lactate level of 2.8 in comparison to survivors (1.4, *p* = 0.076). In the ECMO group, as seen in Figure 3, non-survivors had a higher plasma lactate level prior to ECMO initiation: a median of 5.1 compared to 3.4 for survivors (*p* = 0.041).

### 3.5. Multivariate Analysis 

Multivariate regression across shock stages C, D, and E revealed correlations with mortality among age (*p* = 0.056), BMI (*p* = 0.014), postoperative dialysis (*p* < 0.001), ECMO (*p* = 0.043), and hospital stay (*p* < 0.001). In the non-ECMO versus ECMO group analysis, correlations with mortality were found among age (*p* = 0.005), preoperative creatinine level (*p* = 0.018), kidney injury (*p* = 0.005), ICU stay (*p* = 0.003), and previous cardiac surgery (*p* = 0.029). Kaplan–Meier analysis demonstrated a cumulative survival rate of 43% after 30 days at shock stage D compared to 14% at shock stage E (*p* < 0.001). Additionally, the ECMO patients exhibited a better cumulative survival rate of 38% compared to the cumulative survival rate of 27% of the non-ECMO patients (*p* = 0.006).

## 4. Discussion

ECMO represents an aggressive form of cardiopulmonary support for patients in dire clinical conditions. Based on the SCAI classifications, we attempted to differentiate the severity of cardiogenic shock and the use of ECMO. Our findings indicate that a higher stage of cardiogenic shock is associated with increased mortality, consistent with Jentzer et al.’s findings [17]. They reported that a higher SCAI shock stage correlated with increased in-hospital mortality among non-cardiotomy patients experiencing cardiogenic shock [11,18]. However, they noted that this shock classification could not be applied to patients with PCCS, given the presence of an alternative underlying pathology leading to shock, often compounded by a distributive component. We observed a notable difference between the in-hospital mortality of the non-ECMO and ECMO patients (85% vs. 60%). This could potentially be explained by the circulatory support resulting in the perfusion of end organs and the additional provided time for myocardial recovery [4,5,6,8]. It remains unclear whether the initiation of ECMO directly contributes to the survival of those who undergo it or if other variables, such as the quality of therapy or the severity of the illness, come into play. Moreover, in our results, the patients deteriorating from shock stage D to shock stage E upon ECMO initiation and those receiving ECMO at shock stage E exhibited a mortality rate of 90%. Based on these findings, one might suggest that refraining from ECMO therapy in such situations could be the appropriate course of action. One aspect to consider is that pre- and intraoperative conditions varied among the patients; however, there were no apparent differences in left ventricular ejection fraction, pulmonary artery pressure, or types of interventions between the non-ECMO and ECMO patients. Regarding the postoperative occurrences of left, right, or biventricular failure, we observed no significant differences across the different SCAI shock stages or ECMO subgroups. It is important to emphasize that a significant selection bias exists, as patients deemed to have refractory PCCS were specifically selected for ECMO therapy, and patients who died within 72 h postoperatively were likewise included in the non-ECMO group. 

The most challenging question remains at what point and for which patients initiating ECMO is justifiable. The longer one waits, the greater the risk of missing the window for ECMO initiation, potentially leading to end-organ failure and death. On the contrary, the initiation of invasive and expensive ECMO may occur when it turns out to be unnecessary, and it may even hinder myocardial recovery due to an increased afterload associated with ECMO [4,5]. In comparison to the non-ECMO patients, the ECMO patients in our study were associated more with adverse events, such as the need for dialysis (60% vs. 42%), AMI (27% vs. 19%), and CVA (12% vs. 17%). From our perspective, the observed 25% difference in mortality favoring ECMO patients compared to non-ECMO patients outweighs the difference in adverse events, particularly given the 41-month follow-up period for the survivors of the ECMO group. Nevertheless, ECMO persists as a costly intervention, posing a substantial burden on healthcare systems. This is compounded by the extended ICU stays of ECMO patients, along with the considerable expense associated with ECMO hardware and software. While studies list contraindications, like uncontrolled bleeding, sepsis, aortic valve regurgitation, and kidney injury, these are challenges that can potentially be overcome [4,5,9]. In our study, the most notable distinction between the non-ECMO and ECMO groups was an mean age difference of 8 years. The EACTS/STS/AATS/ELSO expert consensus indicates that age is a relative risk factor, but it is plausible that younger individuals were prioritized for ECMO to afford them an additional opportunity, a consideration that may not have been extended to the older group [5]. Several studies have identified risk factors associated with in-hospital mortality in PCCS patients receiving ECMO, including previous cardiac surgery, age, thoracic aortic operations, and diabetes mellitus [8,10,15,16]. Our findings further confirm this association, as age, preoperative creatinine level, kidney injury, previous cardiac surgery, and ICU stay were identified as risk factors for in-hospital mortality.

Several studies suggest that earlier initiation of ECMO could prevent end-organ failure by ensuring circulatory support, with lactate and VIS playing a crucial role in the decision-making of initiating ECMO [14,19]. Lactate is a proven marker for end-organ perfusion, and a high plasma lactate level is associated with higher in-hospital mortality in PCCS patients receiving ECMO [8,10,15,16]. Our results show that non-survivors in the ECMO group had a median plasma lactate level of 5.1 pre-ECMO initiation, which was higher than the plasma lactate level in the survivors (3.4). This indicates that the non- survivors were in a more compromised clinical condition and that irreversible damage may have already occurred, ultimately resulting in higher plasma lactate levels and mortality, for which ECMO therapy may no longer be effective. Moreover, our results show that more ECMO patients compared to non-ECMO patients had normalized plasma lactate levels within 48 h (25% vs. 4%). This suggests that ECMO provided circulatory support, and thus, possibly prevented end-organ failure. By preventing hypoperfusion of the end organs, we could possibly prevent complications, such as kidney injury and abdominal ischemia, especially because 20% of the patients who did not receive ECMO and subsequently died did so due to the development of sepsis. These patients were mostly held unfavorable due to the fact that sepsis was attributable to abdominal ischemia resulting from the low flow state, and they were no longer compatible with recovery.

An often overlooked aspect in clinical practice is determining the threshold for pharmacological therapy. Exceeding this threshold can lead to myocardial toxicity, cardiac ischemia, and catecholamine-induced metabolic alterations in end organs [19]. The significance of pharmacological therapy, especially inotropes, has been previously outlined by Samuel et al. and validated by subsequent studies [20]. There is a growing emphasis on the early initiation of ECMO and the avoidance of high dosages of vasopressor and inotropes, resulting in improved clinical outcomes. In our study, we observed a median VIS of 32 in the ECMO group just before ECMO initiation, and a gradual decrease over time. Conversely, the non-ECMO group showed the highest VIS (35) 2 days postoperative. Both of these values are lower than the proposed threshold of 38 for initiating ECMO, as suggested in the study by Hyun et al. [14]. However, it is peculiar that the ECMO group required vasopressors and inotropes for a longer duration despite receiving circulatory support (216 h vs. 96 h). Studies have suggested that ECMO initiates systemic inflammatory response syndrome the moment the patient’s blood interacts with the foreign surface of the cannulas and that prolonged ECMO therapy further activates coagulative and inflammatory cascades, contributing to more endothelial injury and end-organ dysfunction [21]. This could be a possible explanation for the prolonged need for vasopressors and inotropes in the ECMO group.

### Limitations

One of the limitations of our study is its retrospective nature, which may have led to missing data and potential bias, making it difficult to perform statistical tests. Additionally, the SCAI classification originated from studies predominantly involving non-surgical patients, resulting in a skewed representation of criteria across the different shock stages. It is worth noting that the classification has limitations, particularly in shock stage E, which includes all deaths due to cardiac disease and may have impacted our results. Moreover, there was a disparity in the timing of the staging of cardiogenic shock. The non-ECMO patients were staged at their highest level of shock, while the ECMO patients were staged just before ECMO initiation. Additionally, we did not examine the role of pre-existing cardiac disease in the development of cardiogenic shock and the efficacy of ECMO therapy. It is possible that in patients with poor cardiac function prior to the surgery, the ability to tolerate any further deterioration or stunning may be limited. Furthermore, the duration of shock may have been overestimated, given that we adhered to the moment when both vasopressors and inotropes were discontinued.

## 5. Conclusions

We believe that ECMO could enhance survival in PCCS patients who would otherwise face dire outcomes. However, pinpointing the ideal timing for ECMO initiation remains a major challenge. Although our study reveals that ECMO led to more rapid normalization of lactate levels, it also indicates that non-survivors compared to survivors in the ECMO group had higher plasma lactate levels prior to ECMO initiation, suggesting the occurrence of irreversible end-organ damage, resulting in higher mortality rates. Nevertheless, interpreting the survival benefit of ECMO therapy is complex due to factors such as selection bias and variations in the timing of shock staging between the ECMO and non-ECMO groups, leading to skewed SCAI shock staging.

## Figures and Tables

**Table 1:** Baseline characteristics of SCAI shock stages.

Variable	Stage C (*n* = 95)	Stage D (*n* = 59)	Stage E (*n* = 66)	Total (*n* = 220)	*p*-Value
Age, years	70 ± 9	66 ± 11	72 ± 8	69 ± 9	0.002
Male (%)	58 (61%)	38 (64%)	38 (58%)	134 (61%)	0.736
BMI	26 ± 4	27 ± 7	27 ± 10	26 ± 7	0.001
NYHA					
Class III	35 (37%)	22 (37%)	22 (33%)	79 (36%)	0.872
Class IV	23 (24%)	18 (31%)	22 (33%)	63 (29%)	0.422
CCS, Class IV	14 (15%)	13 (22%)	20 (30%)	47 (21%)	0.060
Instable angina pectoris	7 (7%)	7 (12%)	12 (18%)	26 (12%)	0.112
Chronic lung disease	12 (13%)	10 (17%)	15 (22%)	37 (17%)	0.242
Arterial vascular pathology	11 (12%)	8 (14%)	15 (22%)	34 (15%)	0.140
Diabetes mellitus	23 (24%)	15 (25%)	31 (47%)	69 (31%)	0.005
Recent myocardial infarction	20 (21%)	21 (36%)	25 (38%)	66 (30%)	0.040
Previous cardiac surgery	17 (18%)	16 (27%)	12 (18%)	45 (20%)	0.332
LVEF					
Normal (>55%)	52 (55%)	30 (51%)	28 (42%)	110 (50%)	0.274
Moderate (40–55%)	32 (34%)	18 (31%)	26 (39%)	76 (35%)	0.572
Poor (25–40%)	8 (8%)	7 (12%)	11 (17%)	26 (12%)	0.292
Very poor (<25%)	2 (2%)	4 (7%)	1 (2%)	7 (3%)	0.183
Pulmonary artery pressure					
Normal (<25 mm Hg)	71 (75%)	41 (69%)	49 (74%)	161 (73%)	0.703
Mild (40–60 mm Hg)	14 (15%)	8 (14%)	15 (22%)	37 (17%)	0.311
Severe (>60 mm Hg)	9 (9%)	10 (17%)	2 (3%)	21 (10%)	0.031
Active endocarditis	5 (%)	2 (3%)	8 (1%)	15 (7%)	0.112
Creatinine, preoperative	99 (79–117)	97 (78–132)	103 (79–128)	99 (78–119)	0.853
Dialysis-dependent, preoperative	1 (1%)	1 (2%)	3 (5%)	5 (2%)	0.323
Critical preoperative condition	16 (17%)	14 (24%)	13 (20%)	43 (20%)	0.577

Note: Values are the median (IQL: interquartile range), mean (± SD: standard deviation), or *n* (%). Statistical significance was determined at the *p* < 0.05 level. BMI, body mass index; NYHA, New York Heart Association; CCS, Canadian Cardiovascular Classification System; LVEF, left ventricular ejection fraction.

**Table 2:** Surgical data of SCAI shock stages.

Variable	Stage C (*n* = 95)	Stage D (*n* = 59)	Stage E (*n* = 66)	Total (*n* = 220)	*p*-Value
**Urgency**					
**Elective**	48 (51%)	28 (47%)	26 (39%)	102 (46%)	0.372
**Urgent**	25 (26%)	12 (20%)	19 (29%)	56 (25%)	0.539
**Emergent**	20 (21%)	16 (27%)	18 (27%)	54 (25%)	0.576
**Salvage**	2 (2%)	3 (5%)	3 (5%)	8 (4%)	0.564
**EuroSCORE II (%)**	7 (3–14)	11 (4–19)	10 (4–18)	8 (4–17)	0.166
**Low risk, 0–4%, pts.**	30 (32%)	17 (29%)	13 (20%)	60 (27%)	0.238
**Intermediate risk, 4–8%, pts.**	24 (25%)	9 (15%)	14 (21%)	47 (21%)	0.338
**High risk, 8%>, pts.**	41 (43%)	33 (56%)	39 (59%)	113 (51%)	0.099
**Surgery**					
**Isolated CABG**	23 (24%)	12 (20%)	18 (27%)	53 (24%)	0.664
**Isolated, non-CABG**	17 (18%)	18 (31%)	7 (11%)	42 (19%)	0.017
**CABG + AVR**	9 (9%)	1 (2%)	14 (21%)	24 (11%)	0.002
**AVR + MVR/P**	3 (3%)	2 (3%)	2 (3%)	7 (3%)	0.993
**Stand-alone and**					
**concomitant mixed**					
**CABG**	51 (54%)	24 (41%)	41 (53%)	116 (53%)	0.055
**AVR**	26 (27%)	18 (31%)	21 (32%)	65 (30%)	0.816
**MVR/P**	31 (33%)	21 (36%)	16 (24%)	68 (31%)	0.348
**Valves**	52 (55%)	34 (58%)	38 (58%)	124 (56%)	0.914
**Aortic**	27 (28%)	15 (25%)	12 (18%)	54 (25%)	0.327

Note: Values are the median (IQL: interquartile range) or *n* (%). Statistical significance was determined at the *p* < 0.05 level. EuroSCORE II, European System for Cardiac Operative Risk Evaluation; CABG, coronary artery bypass grafting; AVR, aortic valve replacement; MVR/P, mitral valve replacement/plasty.

**Table 3:** Baseline characteristics of the non-ECMO and ECMO groups.

Variable	Non-ECMO, Stages D and E (*n* = 52)	ECMO (*n* = 73)	Total (*n* = 125)	*p*-Value
**Age, years**	73 ± 8	65 ± 10	69 ± 10	<0.001
**Male (%)**	31 (60%)	45 (62%)	76 (61%)	0.819
**BMI**	27 ± 9	27 ± 9	27 ± 9	0.848
**NYHA**				
**Class III**	17 (33%)	27 (37%)	44 (35%)	0.620
**Class IV**	21 (40%)	19 (26%)	40 (32%)	0.090
**CCS, Class IV**	18 (34%)	15 (21%)	32 (26%)	0.079
**Instable angina pectoris**	11 (21%)	8 (11%)	19 (15%)	0.118
**Chronic lung disease**	15 (29%)	10 (14%)	25 (20%)	0.037
**Arterial vascular** **pathology**	13 (25%)	10 (14%)	23 (18%)	0.108
**Diabetes mellitus**	27 (52%)	19 (26%)	46 (37%)	0.003
**Neurological dysfunction**	2 (4%)	2 (3%)	4 (3%)	0.729
**Recent myocardial** **infarction**	20 (38%)	26 (36%)	46 (37%)	0.745
**Previous cardiac surgery**	7 (13%)	21 (29%)	28 (22%)	0.043
**LVEF**				
**Normal (>55%)**	20 (38%)	38 (52%)	58 (46%)	0.133
**Mild (40–55%)**	23 (44%)	21 (29%)	44 (35%)	0.074
**Moderate (25–40%)**	8 (15%)	10 (14%)	18 (14%)	0.791
**Severe (<25%)**	1 (2%)	4 (5%)	5 (4%)	0.317
**Pulmonary artery pressure**				
**Normal incl. 25 mm Hg**	40 (77%)	50 (68%)	90 (72%)	0.301
**Mild, >40 mm Hg**	10 (19%)	13 (18%)	23 (18%)	0.840
**Severe, >60 mm Hg**	2 (4%)	10 (14%)	12 (10%)	0.065
**Active endocarditis**	4 (8%)	6 (8%)	10 (8%)	0.915
**Creatinine, preoperative**	102 (78–134)	99 (78–126)	100 (78–130)	0.722
**Dialysis-dependent, ** **preoperative**	2 (4%)	2 (3%)	4 (3%)	0.729
**Critical preoperative** **condition**	11 (21%)	16 (22%)	27 (22%)	0.919

Note: Values are the median (IQL: interquartile range), mean (± SD: standard deviation), or *n* (%). Statistical significance was determined at the *p* < 0.05 level. BMI, body mass index; NYHA, New York Heart Association; CCS, Canadian Cardiovascular Classification System; LVEF, left ventricular ejection fraction.

**Table 4:** Clinical outcomes of the different SCAI shock stages.

Variable	Stage C (=95)	Stage D (*n* = 59)	Stage E (*n* = 66)	Total (*n* = 220)	*p*-Value
Operation time, minutes	291 (207–364)	320 (228–448)	261 (211–347)	285 (212–383)	0.115
Right-sided ventricular failure	31 (33%)	27 (46%)	16 (24%)	74 (34%)	0.038
Left-sided ventricular failure	37 (39%)	26 (44%)	33 (50%)	96 (44%)	0.379
Biventricular failure	27 (28%)	6 (10%)	16 (24%)	49 (22%)	0.049
Kidney injury	25 (26%)	40 (68%)	37 (56%)	102 (46%)	<0.001
Dialysis, postoperative	14 (15%)	35 (59%)	31 (47%)	80 (36%)	<0.001
Diagnostic laparoscopy abdomen	4 (4%)	2 (3%)	4 (6%)	10 (5%)	0.692
CVA	7 (7%)	10 (17%)	8 (12%)	25 (11%)	0.185
Myocardial infarction, peri- or postoperative	16 (17%)	16 (27%)	14 (21%)	46 (%)	0.312
Rethoracotomies, total patients	35 ± 1 24 (25%)	88 ± 2 30 (51%)	35 ± 1 24 (36%)	158 ± 1 78 (35%)	<0.001 <0.001
ECMO	0	53 (90%)	20 (30%)	73 (33%)	<0.001
Pacemaker implantation	5 (5%)	2 (3%)	4 (6%)	11 (5%)	0.782
Postoperative PCI	3 (3%)	10 (17%)	7 (11%)	20 (9%)	0.063
Neurological infaust prognosis	0	4 (7%)	7 (11%)	11 (5%)	0.008

Note: Values are the median (IQL: interquartile range), mean (±SD: standard deviation), or *n* (%). Statistical significance was determined at the *p* < 0.05 level. CVA, cerebral vascular accident; ECMO, extracorporeal membrane oxygenation; PCI, percutaneous coronary intervention.

**Table 5:** Clinical outcomes of the different SCAI shock stages.

Variable	Stage C (*n* = 95)	Stage D (*n* = 59)	Stage E (*n* = 66)	Total (*n* = 220)	*p*-Value
Mortality, in-hospital	0	26 (44%)	62 (94%)	88 (40%)	<0.001
Mortality, out-of- hospital	18 (19%)	8 (14%)	1 (2%)	27 (12%)	<0.001
Follow-up, months	39 (22–54)	40 (23–54)	41 (31–42)	40 (22–54)	0.903
Intensive care unit stay, hours	167 (118–385)	390 (230–760)	122 (53–310)	199 (113–476)	<0.001
Intensive care unit stay, without transfers, hours	167 (115–356)	428 (216–654)	122 (52–308)	178 (94–418)	<0.001
Hospital stay, hours	518 (261–823)	474 (265–1003)	148 (53–330)	350 (200–762)	<0.001
Hospital stay, without transfers, hours	566 (293–839)	468 (258–1001)	138 (52–326)	335 (154–760)	<0.001
Hospital transfers	33 (35%)	15 (25%)	2 (3%)	50 (22%)	<0.001
Lactate normalization, hours <48 h, patients *	27 (22–38) 55 (58%)	27 (19–32) 26 (44%)	31 (28–35) 11 (%)	42 (26–74) 93 (42%)	0.513 <0.001
Lactate normalization without death, hours <48 h, patients *	27 (22–38) 55 (58%)	29 (22–33) 19 (32%)	51 (29–241) 1 (2%)	28 (22–38) 75 (35%)	0.795 <0.001
VIS normalization, hours	96 (72–144)	216 (162–342)	92 (72–192)	120 (72–216)	<0.001
VIS normalization without death, hours	96 (72–144)	216 (120–312)	420 (222–600)	120 (72–168)	<0.001

Note: Values are the median (IQL: interquartile range) or *n* (%). Statistical significance was determined at the *p* < 0.05 level. VIS, vasoactive–inotropic score. * Number of patients reaching normal plasma lactate levels under 2 mmol/L within 48 hours.

**Table 6:** Clinical outcomes of non-ECMO and ECMO patients.

Variable	Non-ECMO, Stage D and E (*n* = 52)	ECMO (*n* = 73)	Total (*n* = 125)	*p*-Value
Mortality, in-hospital	44 (85%)	44 (60%)	88 (70%)	0.003
Mortality, out-of- hospital	2 (4%)	7 (10%)	9 (8%)	0.014
Follow-up, months	38 (22–49)	41 (23–54)	41 (23–54)	0.856
Neurological infaust prognosis	2 (4%)	9 (12%)	11 (9%)	0.099
Right-sided ventricular failure	15 (29%)	28 (38%)	43 (34%)	0.270
Left-sided ventricular failure	24 (46%)	35 (48%)	59 (47%)	0.843
Biventricular failure	12 (23%)	10 (14%)	22 (18%)	0.255
Kidney injury	29 (56%)	48 (66%)	77 (62%)	0.258
Dialysis, postoperative	22 (42%)	44 (60%)	66 (53%)	0.047
Diagnostic laparoscopy abdomen	2 (4%)	4 (5%)	6 (5%)	0.674
CVA	9 (17%)	9 (12%)	18 (14%)	0.435
Myocardial infarction, peri- or postoperative	10 (19%)	20 (27%)	30 (24%)	0.292
Rethoracotomy, total patients	29 ± 1 19 (37%)	94 ± 2 35 (48%)	123 ± 1 54 (43%)	0.007 0.204
Postoperative PCI	3 (6%)	14 (19%)	17 (14%)	0.085
Operation time, minutes	245 (202–325)	320 (234–463)	281 (214–417)	<0.001
Intensive care unit stay, hours	158 (64–339)	317 (142–666)	235 (74–556)	0.019
Intensive care unit stay without transfers, hours	147 (56–334)	308 (103–597)	218 (70–477)	0.052
Hospital stay, hours	198 (64–453)	333 (142–763)	300 (74–649)	0.024
Hospital stay without transfers, hours	162 (56–344)	313 (103–721)	252 (70–585)	0.043
Hospital transfers	6 (12%)	11 (15%)	17 (14%)	0.570
Lactate normalization, hours <48 h *	61 (39–74) 6 (12%)	32 (24–62) 28 (38%)	41 (27–68) 34 (27%)	0.613 <0.001
Lactate normalization without deaths, hours <48 h *	70 (50–112) 2 (4%)	41 (24–62) 18 (25%)	44 (28–69) 20 (16%)	0.222 0.002
VIS normalization, hours	96 (72–198)	216 (120–336)	144 (72–312)	0.005
VIS normalization without deaths, hours	96 (90–228)	216 (168–330)	216 (120–312)	0.179

Note: Values are the median (IQL: interquartile range) or *n* (%). Statistical significance was determined at the *p* < 0.05 level. CVA, cerebral vascular accident; PCI, percutaneous coronary intervention; VIS, vasoactive–inotropic score. * Number of patients reaching normal plasma lactate levels under 2 mmol/L within 48 h.

**Table 7:** ECMO-related settings.

Variable	Intraoperative ECMO (*n* = 26)	Postoperative ECMO (*n* = 47)	Total (*n* = 73)	*p*-Value
Central cannulation	13 (50%)	25 (53%)	38 (52%)	0.723
Peripheral cannulation	20 (77%)	39 (83%)	59 (81%)	0.405
Central and peripheral cannulation	18 (69%)	8 (17%)	26 (36%)	<0.001
ECMO implantation after surgery, hours	0	22 (10–47)	9 (0–24)	<0.001
ECMO mean flow, liter per minute	3.8 ± 0.7	3.8 ± 0.5	3.8 ± 0.6	0.904
ECMO duration, hours	118 (56–218)	121 (51–175)	118 (52–176)	0.587
ECMO duration without deaths, hours	118 (83–239)	127 (97–168)	124 (95–175)	0.688

Note: Values are the median (IQL: interquartile range), mean (±SD: standard deviation), or *n* (%). Statistical significance was determined at the *p* < 0.05 level. ECMO, extracorporeal circulatory membrane oxygenation.

## Data Availability

The datasets generated or analyzed during this study are available from the corresponding author upon reasonable request.

## Supplementary Materials

The following supporting information can be downloaded at: https://www.mdpi.com/article/10.3390/jcm13072118/s1, Table S1: Definitions of the secondary outcomes used by the Dutch Heart Registration (NHR). Table S2: Surgical data of the non-ECMO and ECMO group. Figure S1: (A) Median VIS and plasma lactate levels after primary surgery of the non-survivors of non-ECMO patients, including only stage D and E. *Notes* OR, operation room; (B) Median VIS and plasma lactate levels after primary surgery of the survivors of non-ECMO patients.
