# Supplementary material for: Comparative Analysis of Therapeutic Strategies in Post-Cardiotomy Cardiogenic Shock: Insight into a High-Volume Cardiac Surgery Center
Source: J Clin Med. 2024 Apr 5;13(7):2118. doi: 10.3390/jcm13072118 (PMC11012770; doi:10.3390/jcm13072118)
Supplement: Supplementary file 1 [file jcm-13-02118-s001.zip › jcm-2911104-supplementary.pdf]

Journal of
Clinical Medicine

MDPI

Supplementary Materials

Methods

Table S1 Definitions of the secondary outcomes used by the Dutch Heart Registration (NHR).

Variable	Definition
New York Heart Association (NYHA) functional classification for heart failure	This is as follows, and the highest class within the 2 weeks preceding the current intervention was provided:<br>- Class I: No symptoms during ordinary physical activity.<br>- Class II: Symptoms with ordinary exertion.<br>- Class III: Symptoms with less than ordinary exertion.<br>- Class IV: Symptoms at rest.
Canadian Cardiovascular Classification (CCS), class IV	The patient has angina pectoris classified as Class IV according to the CCS. This is characterized by the inability to perform any physical activity without the occurrence of angina pectoris or experiencing angina pectoris at rest.
Chronic lung disease	Prolonged use of bronchodilators or steroids due to lung disease.
Arterial vascular disease	If one or more of the following criteria are met:<br>- Intermittent claudication<br>- Carotid occlusion or > 50% stenosis<br>- Amputation due to arterial disease<br>- Previous or planned surgery on the abdominal aorta, arteries of the limbs, or carotids.
Neurological dysfunction	A disease that significantly restricts ambulation or daily functioning.
Previous cardiac surgery	Previous heart surgery involving the opening of the pericardium, including transapical transcatheter heart interventions (THIs).
Left ventricular ejection fraction (LVEF)	The percentage of end-systolic volume of blood in the left ventricle relative to the end-diastolic volume, known as the relative stroke volume. Utilizing the most recent determination documented in a diagnostic report prior to the intervention. Note: The recorded ejection fraction (EF) should not have been established more than 3 months before the intervention. Preferably provide the precisely calculated EF. If a percentage range is reported, the average of this range is submitted (e.g., 50-55% should be submitted as 53%). If only a descriptive value is recorded (e.g., moderate), the corresponding percentage from the list is provided: Good = 55%, Moderate = 40%, Poor = 25%, Very poor = 20%.
Pulmonary artery pressure (PAP)	The systolic pressure in the pulmonary artery in mmHg measured through invasive pressure monitoring or estimated through an echo prior to the current intervention. Preferably provide the precisely measured pressure value. If only a descriptive value is recorded for pulmonary hypertension (e.g., moderate), provide the corresponding pressure values from the list below: Normal pressure = 25 mmHg, moderately elevated pressure = 40 mmHg, severely elevated pressure = > 60 mmHg.
Active endocarditis	At the time of the intervention, the patient is still undergoing treatment with an antibiotic due to endocarditis.
Critical preoperative condition	If one or more of the following criteria are present:<br>- Preoperative ventricular tachycardia or fibrillation at the start of the intervention, preoperative sudden death survivor, or preoperative resuscitation.<br>- Preoperative mechanical ventilation before arrival in the operating room.

	- Preoperative administration of inotropes.<br>- Preoperatively inserted intra-aortic balloon pump (IABP).<br>- Preoperatively diagnosed renal failure (anuria or oliguria < 10 ml/hour).
Instable angina pectoris	Angina pectoris requiring intravenous nitrate therapy up to and including the arrival in the operating room for the current intervention.
Recent myocardial infarction	Having experienced a myocardial infarction in the 90 days preceding the current intervention.
Dialysis, preoperative	Hemodialysis or peritoneal dialysis due to continuous renal failure at the time of admission for the current intervention. Continuous veno-venous hemofiltration (CVVH) due to renal failure is also included (excluding cases where it is temporary and only used for fluid removal in heart failure).
Dialysis, preoperative	Hemodialysis or peritoneal dialysis due to continuous renal failure at the time of admission for the current intervention. Continuous veno-venous hemofiltration (CVVH) due to renal failure is also included (excluding cases where it is temporary and only used for fluid removal in heart failure).
Urgency: elective, urgent, emergent, saving	The status of the current intervention is subdivided into:<br>- Elective: Standard admission for the operation.<br>- Urgent: Patients who were not admitted electively for surgery but require an intervention for medical reasons during the current admission. These patients cannot be discharged home without a definitive procedure.<br>- Emergency: Unplanned intervention that, for medical reasons, cannot wait until the beginning of the next working day.<br>- Rescue: Patients requiring cardiopulmonary resuscitation (external cardiac massage) en route to the operating room or prior to the administration of anesthesia. This does not include cardiopulmonary resuscitation that occurs after the administration of anesthesia.
EuroSCORE II, categories	Low risk patient is defined as 0 to 4 percent, medium risk falls between 4 to 8 percent, and high risk is considered to be 8 percent and above.
Interventions	The weight assigned to the current planned intervention is based on major procedures, with the following categories:<br>Isolated CABG:<br>Intervention consisting solely of a Coronary Artery Bypass Grafting (CABG) and no other major cardiac procedure.<br>1 Procedure (non-CABG):<br>Intervention involving a single major procedure that is not CABG. Examples include aortic valve replacement, correction of septal defect, or other similar procedures.<br>2 Procedures:<br>Intervention comprising two major procedures. Examples include CABG + aortic valve replacement (AVR), CABG + mitral valve repair (MVR), Aortic Valve Surgery + Replacement of Ascending Aorta, CABG + Maze Procedure, Aortic Valve Surgery + MVR, etc.<br>3 or more procedures:<br>Intervention involving three or more major procedures. Examples include AVR + MVR + CABG, AVR + MVR + CABG, MVR + CABG + Tricuspid valve annuloplasty, etcetera.<br>Only major cardiac procedures contribute to the total count. Examples of procedures not considered major include thoracatomy, closure of the sternum, myocardial biopsy, insertion of IABP (Intra-Aortic Balloon Pump), leads, additional placement of a pacemaker and/or ICD (Implantable

	Cardioverter Defibrillator), closure of aortotomy, closure of atriotomy, removal of the left atrial appendage, coronary endarterectomy as part of a CABG, etcetera.
Kidney injury	Renal failure is considered if during the postoperative period one or more of the following STS criteria are met:<br>- Renal replacement therapy (dialysis, continuous veno-venous hemofiltration - CVVH) that was not present preoperatively.<br>- Highest postoperative creatinine value > 177 mmol per liter and doubling of the preoperative value (with the preoperative value being the creatinine level used for calculating the EuroSCORE).
Myocardial infarction, peri-or postoperative	A myocardial infarction occurring after the current intervention (including periprocedural myocardial infarctions, type 5 myocardial infarctions that occur within 48 hours of the intervention): the standard measurement of biomarkers within 48 hours after the intervention is a requirement.
Neurological infaust prognosis	A neurologist has determined that a postoperative cerebrovascular accident (CVA) occurred during the hospitalization of the current intervention (excluding transient ischemic attack. CVA is defined as a neurologist-confirmed permanent neurological dysfunction resulting from focal ischemia of the brain, spinal cord, or retina. This ischemia is caused by an acute infarct of neurological tissue due to thrombosis, embolism, systemic hypoperfusion, or bleeding.

Results

Table S2 Surgical data of the non-ECMO and ECMO group.

Variable	Non-ECMO, Stage D and E (*n* = 52)	ECMO (*n* = 73)	Total (*n* = 125)	*p*-Value
EuroSCORE II (%)	10 (5-17)	10 (3-19)	10 (4-18)	0.766
Low risk, pts.	9 (17%)	21 (29%)	30 (24%)	0.139
Intermediate risk, pts.	10 (19%)	13 (18%)	23 (18%)	0.840
High risk, pts.	33 (63%)	39 (53%)	72 (58%)	0.263
Surgery				
CABG + AVR	10 (19%)	8 (11%)	18 (14%)	0.194
AVR + MVR/P	2 (4%)	3 (4%)	5 (4%)	0.941
Stand-alone and concomitant				
CABG	34 (65%)	31 (42%)	65 (52%)	0.011
AVR	14 (3%)	25 (34%)	39 (32%)	0.384
MVR/P	14 (3%)	23 (32%)	37 (30%)	0.580
valves	29 (56%)	43 (59%)	72 (58%)	0.727
aortic	5 (10%)	22 (30%)	27 (22%)	0.006
One intervention				
isolated CABG	14 (27%)	16 (22%)	30 (24%)	0.518
isolated, non-CABG	7 (13%)	18 (25%)	25 (20%)	0.123
Two interventions	24 (46%)	26 (36%)	75 (60%)	0.236
Three or more interventions	7 (13%)	13 (18%)	20 (16%)	0.514

Note: Values are the median (IQL: interquartile range) or *n* (%). Statistical significance was determined at the $p < 0.05$ level. EuroSCORE II, European System for Cardiac Operative Risk Evaluation; CABG, coronary artery bypass grafting; AVR, aortic valve replacement; MVR/P, mitral valve replacement/plasty.

A)

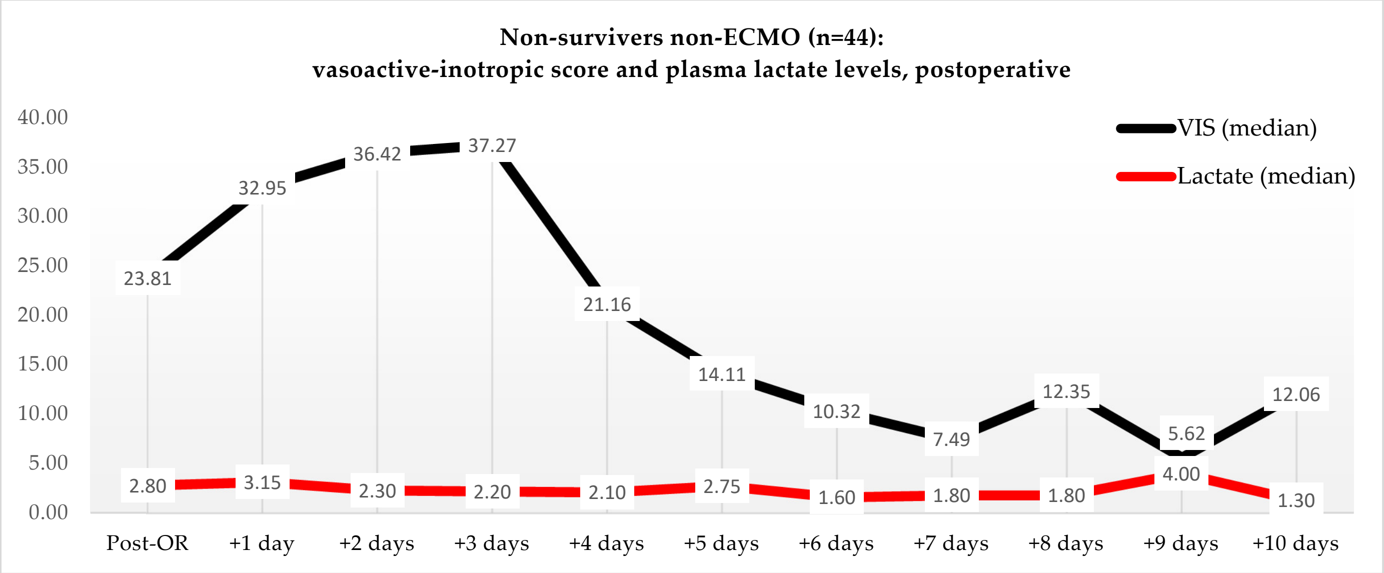

B)

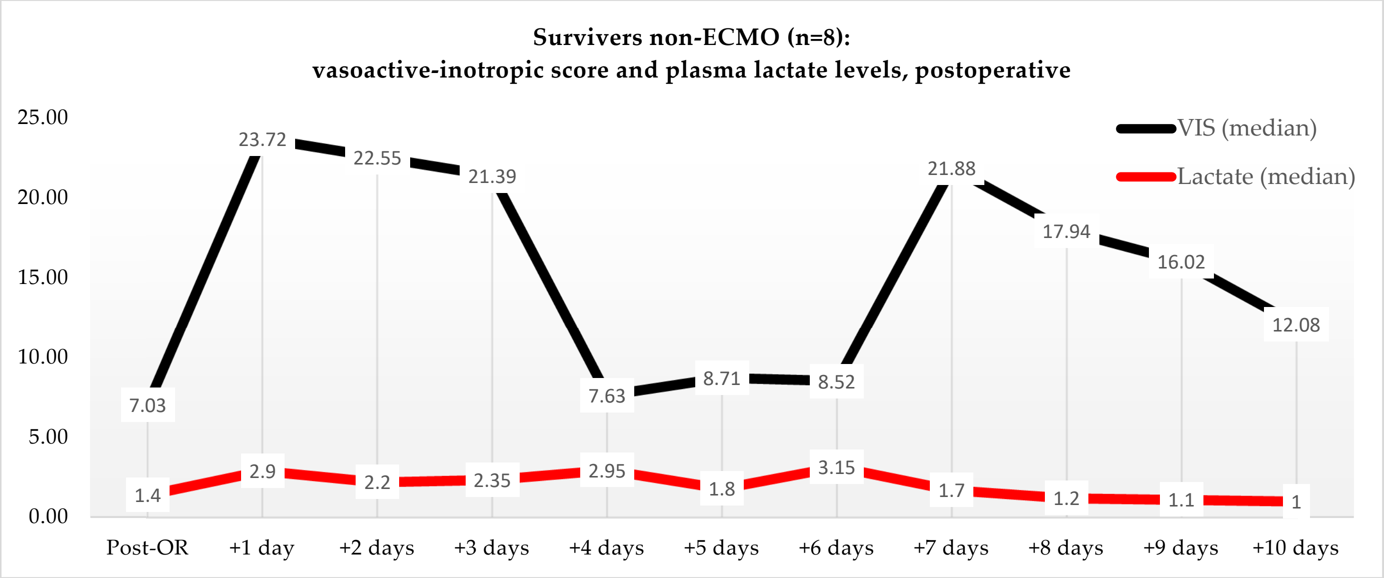

Figure S1 (**A**) Median VIS and plasma lactate levels after primary surgery of the non-survivors of non-ECMO patients, including only stage D and E. Note: OR, operation room. (**B**) Median VIS and plasma lactate levels after primary surgery of the survivors of non-ECMO patients.
